# The physiologic responses to epinephrine during cooling and after rewarming in vivo

**DOI:** 10.1186/cc10465

**Published:** 2011-09-23

**Authors:** Torkjel Tveita, Gary C Sieck

**Affiliations:** 1 Department of Physiology & Biomedical Engineering, Mayo Clinic College of Medicine, Rochester, Minnesota 55905, USA; 2 Anesthesia and Critical Care, Institute of Clinical Medicine, University of Tromsø, N-9037 Tromsø, Norway

## Abstract

**Introduction:**

The purpose of our study was to determine whether hypothermia has any effects on physiological hemodynamic responses to epinephrine (Epi), and whether rewarming reverses these effects.

**Methods:**

Sprague-Dawley rats were instrumented to measure mean arterial pressure (MAP), and left ventricular (LV) pressure-volume changes were recorded by using a Millar pressure-volume conductance catheter. Core temperature was reduced from 37°C to 28°C and returned to 37°C by using both internal and external heat exchangers. Two groups of rats were infused with either saline (*n *= 7), or Epi 0.125 μg/min continuously (*n *= 7). At 33°C, 30°C, and 28°C, the Epi infusion was temporarily increased from 0.125 to 1.25 μg/min.

**Results:**

Before cooling, Epi infusion in both groups resulted in a significant, dose-dependent increase in heart rate (HR), stroke volume (SV), cardiac output (CO), LV dP/dt_max _(maximum derivative of systolic pressure over time), but only Epi infusion at 1.25 μg/min caused elevation of MAP. During cooling to 30°C, Epi infusion at 0.125 μg/min caused a significant elevation of central hemodynamic variables, whereas MAP remained unchanged. In contrast, Epi infusions at 1.25 μg/min caused a significant elevation of MAP during cooling to 28°C but no increases in central hemodynamics. After rewarming, all hemodynamic variables returned to baseline in both groups, but only the saline-treated animals displayed the prehypothermic hemodynamic dose responses to Epi infusions.

**Conclusions:**

This study shows that hypothermia causes a change in the physiological hemodynamic response to Epi, which is not reversed by rewarming.

## Introduction

Guidelines for using inotropic drugs to support cardiovascular function at low core temperatures are not well characterized. Such guidelines are essential for treating patients in acute heart failure, as will be the case during induced therapeutic hypothermia, as well as during rewarming from accidental hypothermia. Detailed knowledge of temperature-dependent changes in pharmacodynamics and pharmacokinetics of such cardioactive drugs is essential for establishing treatment guidelines.

Over the last decade, induced hypothermia is being increasingly used to reduce cerebral damage in patients after resuscitation from sudden cardiac arrest [1,2]. However, after return of spontaneous circulation (ROSC), these patients often have acute heart failure and need inotropic cardiac support to resume adequate circulatory function [1] after induction of hypothermia in which the core temperature is deliberately lowered to 34°C - 32°C and maintained for 24 to 48 hours.

Rewarming shock is a clinically descriptive term that refers to a pathophysiologic state of cardiovascular collapse taking place during or after rewarming from accidental hypothermia [3]. Rewarming shock is recognized as a progressive reduction of cardiac output (CO) and a sudden decrease in arterial blood pressure. To treat or prevent rewarming shock, cardioactive inotropic drugs are commonly necessary to elevate low CO.

Relatively few studies have explored the pharmacologic effects of epinephrine or norepinephrine treatment during hypothermia. Studies on the effects of epinephrine (Epi) used to convert hypothermic cardiac arrest to ROSC during cardiopulmonary resuscitation (CPR) in pigs show diverse effects. In clinical practice, it is recognized that in the acutely failing heart postoperatively or after resuscitations, only drugs such as Epi and NE provide positive inotropy and perfusion pressure. The American Heart Association (AHA) recommends the following algorithm for the use of Epi during hypothermia; at less than 30°C, i.v. epinephrine should not be given, but at greater than 30°C, epinephrine should be given, if indicated, but at longer than standard intervals [4].

In our laboratory, we found that Epi, if given in doses (1.25 μg/ml) that increased CO, without affecting vascular resistance during normothermia, gave rise to vasoconstriction (increased α-adrenoceptor response) but failed to elevate low CO (decreased β-adrenoceptor response) when given during hypothermia [5,6]. Thus, in an attempt to elevate CO during cooling to 28°C in our experimental hypothermia model, the effects of two different Epi doses, 0.125 or 1.25 μg/ml, which both exert significant cardiovascular effects during normothermia, were tested during hypothermia.

## Materials and methods

The investigation conformed to the *Guide for Care and Use of Laboratory Animals*, published by the U.S. National Institutes of Health (NIH Publication No. 85-23, revised 1996) and was performed with the approval of the local Institutional Animal Care and Use Committee.

Sprague-Dawley rats (males, 250 to 300 g), were used in the experiments. Anesthesia was induced by sodium pentobarbital (60 mg/kg body weight, i.p.), followed by a continuous infusion of 7.5 mg/kg/h through an i.v. line in the right jugular vein extended to the right auricle. The animals were monitored for any sign of discomfort during hypothermia and rewarming, so that an additional anesthesia might be provided if necessary.

### Core cooling and rewarming

Animals were cooled and rewarmed by circulating cold or warm water (thermostated water bath type RTE-110; Neslab Instruments, Newington, NH, USA), respectively, through a U-shaped polyethylene tube placed in the lower bowels. In addition, the double-layered operating table made of hollow aluminum was circulated by water from this bath. Core temperature was continuously monitored by using a thermocouple wire, with which the sensor tip was positioned in the lower one third of the esophagus (Thermoalert TH-5; Columbus Instruments, Columbus, OH, USA).

### Hemodynamic measurements

A microtip (2F), P-V catheter (SPR-838, Millar Instruments; Houston, TX, USA) was inserted into the right carotid artery and advanced into the LV under pressure control. In addition, a pressure transducer was connected to a fluid-filled catheter (22 G) implanted in the left femoral artery for recording mean arterial pressure (MAP). LV pressure and volume and MAP signals were digitized at 1 kHz, and by an ARIA P-V conductance system (Millar Instruments) coupled to a PowerLab/4SP A/D converter (AD Instruments, Mountain View, CA, USA) and a PC. HR, maximal LV systolic pressure (LVSP), LV end-diastolic pressure (LVEDP), maximal slope of LV systolic pressure increment (LV dP/dt_max_) and diastolic pressure decrement (LV dp/dt_min_), time constant of LV pressure decay (τ), stroke volume (SV), end-diastolic volume (EDV), cardiac output (CO), stroke work (SW), and MAP were computed by using a cardiac P-V analysis program (PVAN 3.2; Millar Instruments, Houston, TX, USA).

### Special modifications related to the use of conductance measurement and adjustments for parallel volume determination at low core temperatures

This topic was described in detail previously [7]. In brief, the measured conductance must be corrected for parallel conductance induced by the alternating current passing through the blood into the surrounding LV wall or interventricular septum. Parallel conductance is usually measured by the saline bolus injection at the end of experiment. However, difficulties were anticipated when applying this method to our experimental protocol, which required measurements of left ventricular volume at the specific experimental temperature (37°C, 33°C, 30°C, and 28°C). In light of temperature-dependent changes in viscosity of blood, the bolus injection performed at the end of experiment cannot represent the true parallel conductance at the other experimental temperatures. For this reason, parallel conductance was not included in our whole-volume measurements.

For a more accurate assessment of the left ventricular volume, the cuvette calibration method was used, requiring insulator-type cuvettes of known diameter (2, 3, 4, 5, 6, and 7 mm) filled with heparin-treated blood. The volume cuvette containing heparin-treated blood was positioned on the inside of a thermo-controlled water circulator so that the temperature of the blood could be adjusted during the calibration. Considering temperature-dependent viscosity of blood, the volumes measured at our specific experimental temperature should be corrected by using the cuvette-calibration method. Slopes and y-intercepts obtained at these temperatures were applied to the analysis software (PVAN) to convert conductance units to true volume units (μl).

### Experimental protocol

After surgery, the animals were allowed to rest for 60 minutes before the start of the experiment. Based on results from a dose-finding group (*n *= 3), the animals were divided into two groups: one intervention group (*n *= 7), which was continuously infused with Epi, 0.125 μg/min, and intermittently infused with Epi, 1.25 μg/min, at 33°C, 30°C, and 28°C, and one control group (*n *= 7) receiving equivalent volumes of saline during cooling to 28°C. Infusions were terminated at 28°C before rewarming was started.

In both groups, the cardiovascular effects of infusing Epi 0.125 and Epi 1.25 μg/min were tested before cooling as well as after rewarming was completed.

### Statistical analyses

Results are presented as mean ± SEM. For within-group comparisons, data were assessed with one-way ANOVA for repeated measurements followed, if the *F *value was greater than critical, by the Dunnett test. To evaluate differences between groups, one-way ANOVA was used, followed, if the *F *value was greater than critical, by the Tukey test. Differences were considered significant at *p *< 0.05.

## Results

All animals survived cooling and rewarming. Except for single ectopic ventricular beats, no episodes of other arrhythmias occurred during cooling and rewarming.

### Assessment of Epi dose response

**Figure 1:**
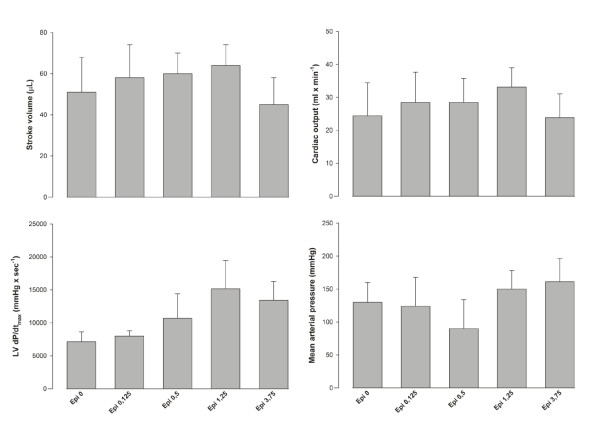
**Dose-finding group (*n *= 3)**.

During normothermia (*n *= 3) (Figure 1), positive cardiac inotropic effects were measured when infusing Epi at doses 0.125 to 1.25 μg/ml, whereas Epi 3.75 μg/ml showed negative effects on cardiac function simultaneous with an increase in afterload. Similar dose-dependent effects of Epi on cardiovascular responses have been shown in a previous study in our laboratory [6].

Hemodynamic stability, up to 5 hours at 37°C, has been documented in numerous studies using the present intact rat model [5-7].

### Baseline 37°C control values

**Figure 2:**
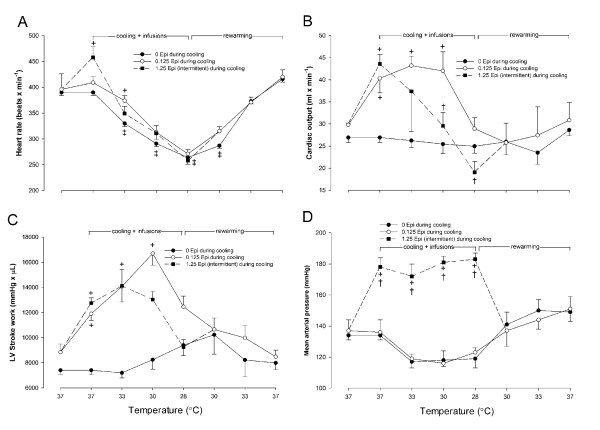
**Heart rate **(a)**, cardiac output **(b)**, left ventricular stroke work **(c)**, and mean arterial pressure **(d) **expressed as a consequence of cooling to 28°C and rewarming in animals receiving saline infusion (*n *= 7), or continuous infusion of (0.125 μg/min), and intermittent infusion of Epi (1.25 μg/min) during cooling to 28°C (*n *= 7)**. Before rewarming, infusions were terminated. Values are expressed as mean ± SEM. **^+^***P *< 0.05 compared with saline group, **†***P *< 0.05 comparisons within Epi group at each temperature, and ‡*P *< 0.05 compared with prehypothermic value in saline group.

**Figure 3:**
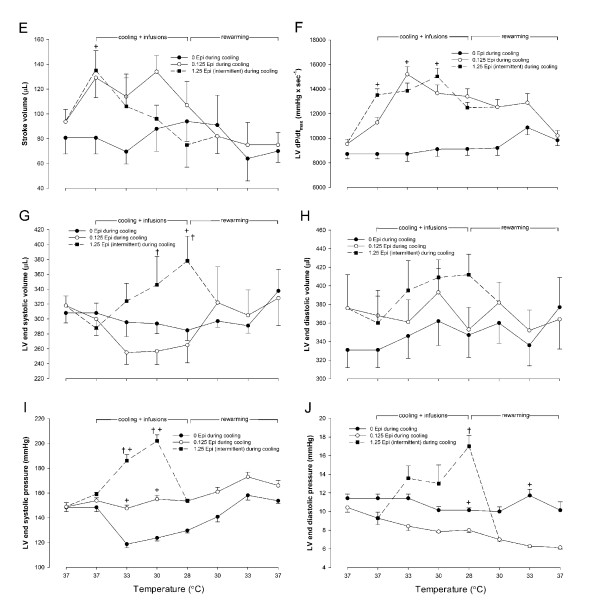
**Stroke volume **(e)**, left ventricular dP/dt_max _**(f)**, left ventricular end-systolic volume **(g)**, left ventricular end-diastolic volume **(h)**, left ventricular end-systolic pressure **(i)**, and left ventricular end-diastolic pressure expressed as a consequence of cooling to 28°C and rewarming in animals receiving saline infusion (*n *= 7), or continuous infusion of Epi (0.125 μg/min), and intermittent infusion of Epi (1.25 μg/min) (*n *= 7)**. Before rewarming, infusions were terminated. Values are expressed as mean ± SEM. **^+^***P *< 0.05 compared with saline group, and **†***P *< 0.05 comparisons within Epi group at each temperature.

No significant differences in baseline values were found for any of the hemodynamic variables measured between saline and Epi groups at 37°C (saline versus Epi group) (Figures 2 and 3).

#### Epinephrine, 0.125 μg/min, versus saline control group

In response to the low-dose (0.125 μg/min) Epi infused at 37°C, CO and SW increased significantly (*P *< 0.05) when compared with saline-treated controls.

#### Epinephrine, 1.25 μg/min, versus saline control group

Treatment with a higher dose (1.25 μg/min) of Epi infused at 37°C caused a significant (*P *< 0.05) increase in HR, SV, CO, SW, and LV dP/dt_max_. MAP increased significantly compared with both saline-treated controls and those with the infusion of Epi 0.125, μg/min.

### Hemodynamic responses to cooling/rewarming

#### Saline control group

In response to cooling, a significant (*P *< 0.05) decrease was noted in HR and Tau (not shown) at temperatures less than 33°C, whereas the other hemodynamic variables remained unchanged during cooling to 28°C and rewarming in this group (Figures 2 and 3).

#### Epi, 0.125 μg/min, or Epi, 1.25 μg/min, versus saline control

At 33°C, infusion of Epi at 0.125 μg/min significantly (*P *< 0.05) increased HR, whereas Epi infusion at 1.25 μg/min did not. In contrast to Epi infusion at 1.25 μg/min, Epi infusion at 0.125 μg/min induced a significant (*P *< 0.05) increase in LV dP/dt_max _and CO, the latter most likely due to the increase in HR at this temperature. Left ventricular end-systolic pressure (LVESP) and SW increased significantly (*P *< 0.05) in response to both doses of Epi at 33°C.

During cooling to 30°C and 28°C, no differences were observed in HR between groups, but the differences in hemodynamic responses between the two doses of Epi became manifest at 30°C, at which Epi infusion at 0.125 μg/min significantly (*P *< 0.05) increased SV and CO when compared with both Epi infusion at 1.25 μg/min and saline infusion. In response to Epi infusion at 0.125 μg/min, SV tended to increase, but this change was not significant. At 30°C, CO was significantly (*P *< 0.05) lower during Epi infusion at 1.25 μg/min than during Epi infusion at 0.125 μg/min. Further, Epi infusion at 1.25 μg/min increased LV end-systolic pressure as well as LV end-systolic volume (LVESV) to significantly (*P *< 0.05) higher levels than did Epi 0.125 μg/min infusion, and this linear increase in LVESV continued during cooling to 28°C.

At 28°C, Epi infusions did not increase SV or CO when compared with the saline group. No change in LV end-diastolic volume was observed within groups or between groups throughout the experiments, a finding that indicates why SV did not increase in response to the Epi 1.25 μg/min infusion. In contrast, the Epi 0.125 μg/min infusion maintained a low LV end-systolic volume throughout cooling to 28°C (demonstrating the positive cardiac inotropic effects of this low-dose Epi on cardiac function at temperatures between 33°C and 30°C). However, at 28°C, the positive inotropic effect of Epi, 0.125 μg/min, to increase CO was no longer present.

Compared with their prehypothermic control values, no statistically significant changes were observed in any of the hemodynamic variables in the two groups during rewarming. During rewarming from 28°C, HR increased linearly and was not significantly lower than prehypothermic control values after 33°C (Figure 2). Likewise, Tau returned to within control values after 30°C rewarming (not shown).

### Posthypothermic responses to Epi

**Figure 4:**
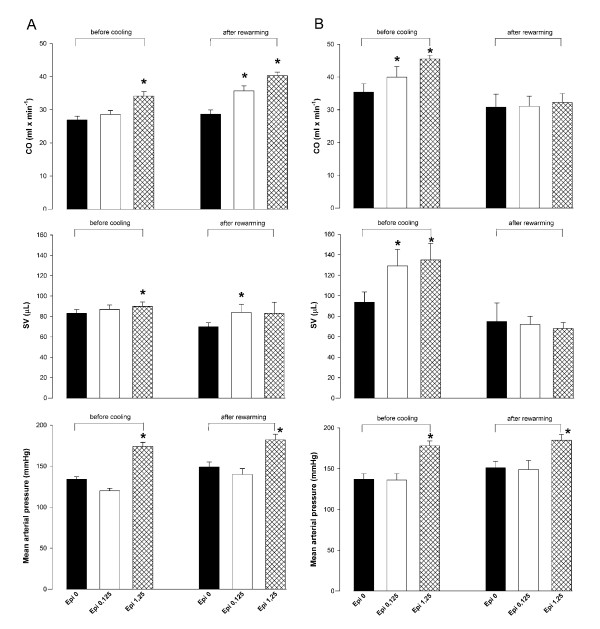
**Changes in cardiac output (CO), stroke volume (SV), or mean arterial pressure in response to Epi, 0.125 μg/min, or Epi, 1.25 μg/min, before cooling and after rewarming in the group receiving saline **(a)**, or Epi **(b) **during cooling to 28°C**. *P < 0.05 compared with corresponding baseline value.

Whereas Epi infusion demonstrated a significant dose/response effect on vascular and LV cardiac function before cooling, this dose/response effect of Epi infusion on LV cardiac function was lost after rewarming (Figure 4). However, in the saline group, the cardiovascular response to Epi infusion was maintained after rewarming.

## Discussion

The present study showed that a low-dose Epi managed to maintain positive inotropic effects on LV cardiac function during cooling to 30°C, but these effects vanished during cooling to 28°C. Second, a higher dose (×10) Epi did not increase SV or CO at these low temperatures, but induced a significant increase in afterload. Third, the prehypothermic dose-dependent increase in LV function in response to both a low- and a high-dose Epi was reproducible after rewarming in the saline control group after cooling and rewarming, but not so in the Epi-treated group.

The results of the present study showing a lack of cardiac inotropic effects, combined with potentiating vascular vasoconstriction at a higher-dose Epi during cooling to 28°C, are in accordance with results previously reported by others [8,9]. However, new information in the present study is that a low-dose Epi exerts positive inotropic effects on cardiac function without affecting afterload during cooling to 30°C, a finding of importance for induced hypothermia, as applied to reduce cerebral damage in patients after resuscitation from sudden cardiac arrest. Further, the lack of cardiac effects of Epi, irrespective of dose, at temperatures below 30°C is of importance for resuscitation from accidental hypothermia.

In mammals, epinephrine is the prominent catecholamine affecting cardiac contractility, acting through stimulation via sarcolemmal β-adrenoceptors. In the myocyte, Epi stimulates β-adrenoceptors, causing phosphorylation of the sarcolemmal L-type Ca^2+ ^channel via cyclic AMP and protein kinase A pathways. This phosphorylation increases the open probability of the channel [10], allowing for greater trans-sarcolemmal Ca^2+ ^influx with each depolarization and producing, in part, the positive inotropic effect of Epi. Farrell et al. [11] reported increased adrenergic (Epi) sensitivity in fish cardiomyocytes during acute cooling by showing that sarcolemmal Ca^2+ ^flux through the L-type Ca^2+ ^channel (I(Ca^2+^)) was significantly more sensitive to adrenergic stimulation during cooling. Thus, although adrenergic stimulation may affect the relative importance of SR Ca^2+ ^release in E-C coupling, temperature-induced modulation of β-adrenoceptors may alter the efficacy of adrenergic effects. If cardiomyocytes from fish acclimatized at 14°C were exposed to 7°C, a significant decrease in I(Ca^2+^) was measured, but if Epi were added, I(Ca^2+^) increased by a factor of 7.2, indicating a significant increase in adrenergic sensitivity in response to cooling [12]. As already mentioned, we found in our laboratory that Epi, when given during normothermia in doses that increased CO without affecting vascular resistance, gave rise to vasoconstriction (increased α-adrenoceptor response), but failed to elevate low CO (decreased β-adrenoceptor response) when given during hypothermia [6]. This finding also fit the idea that cooling may induce a transition from β- to α-adrenoceptor properties, as suggested in a study using isolated rat atria [13]. As a result, Epi may not be an ideal inotropic agent for enhancing CO at low core temperature, but based on observations in the present study, the Epi dose applied during hypothermia must be significantly lower than doses normally used at 37°C core temperature. Further, studies on the effects of Epi used to convert hypothermic cardiac arrest to ROSC during CPR in pigs show diverse effects. These studies all show that Epi will increase coronary blood pressure (CPP) below 30°C, which may be seen as a sign of adequate vascular response to Epi at low core temperatures, but cardiac effects (ROSC) are not conclusive [14-17].

Experimental data from Chernow [18] show that the sympathetic nervous system could be "switched off" at a threshold temperature about 29°C, and hypotensive patients with temperatures below this may benefit from infusions of exogenous catecholamines. In support of this notion, a study on human skin artery responses to tissue cooling showed an increased sensitivity in both α_1_- and α_2_-adrenoreceptors to norepinephrine at 24°C [19]. In addition, if CO could be elevated pharmacologically, rewarming by any means would become more efficient [20]. Danzl and Pozos [21] recommend infusion of low doses of catecholamines in patients who have lower blood pressure than would be expected for that degree of hypothermia and who are not responding to crystalloids and rewarming. However, in the present experiment, we observed that in animals having received Epi during cooling, the physiologic responses to Epi had vanished after rewarming. This finding indicates that the combined impact of hypothermia and adrenergic stimulation has serious impact on β-adrenoceptor function after normothermia is reestablished. Taken together, the use of cardioactive drugs during hypothermic conditions remains quite contradictory. Therefore, pharmacologic treatment applied during the two clinically challenging modalities outlined, induced and accidental hypothermia, call on written treatment protocols or guidelines which are so far largely missing or at least not properly updated. More research is needed to explore temperature-dependent changes in pharmacodynamics and pharmacokinetics of cardioactive drugs to write these guidelines.

## Conclusions

Results from the present study show that a low-dose Epi, in essential contrast to a high-dose Epi, exerts positive inotropic effects during cooling to 30°C. Because of significant hypothermia-induced alterations of cardiac as well as vascular adrenoceptor sensitivity, the use of cardioactive agents not affecting these receptor systems is advised during hypothermic conditions.

## Key messages

• Dose-dependent physiologic responses to epinephrine (Epi) are altered by hypothermia.

• A low-dose Epi may have positive inotropic cardiac effects during cooling to 30°C.

• A high-dose Epi will elevate cardiac afterload substantially but leave cardiac inotropy unchanged.

• At less than 30°C, physiologic inotropic cardiac responses to epinephrine (Epi) are not present.

• If Epi is administered during hypothermia, dose-dependent physiologic responses to Epi are altered after rewarming.

## Abbreviations

CO: cardiac output; dP/dt_max_: maximum derivative of systolic pressure over time; EDP: end-diastolic pressure; EDV: end-diastolic volume; ESP: end-systolic pressure; Epi: epinephrine; ESV: end-systolic volume; HR: heart rate; LV, left ventricular; MAP: mean arterial pressure; SV: stroke volume; SW: stroke work; Tau: isovolumetric relaxation time.

## Competing interests

The authors declare that they have no competing interests.

## Authors' contributions

TT conducted the experiments and processed the hemodynamic data. GCS provided experimental animals as well as all laboratory facilities. TT and GCS finalized the experimental protocol and wrote and approved the final manuscript.
